# Supplementary material for: Evaluation of systemic inflammatory response and lung injury induced by Crotalus durissus cascavella venom
Source: PLoS One. 2020 Feb 21;15(2):e0224584. doi: 10.1371/journal.pone.0224584 (PMC7035002; doi:10.1371/journal.pone.0224584)
Supplement: Attachment — Submitted filename: Comments to the Author.docx [file pone.0224584.s001.docx]

Comments to the Author

The study " EVALUATION OF SYSTEMIC INFLAMMATORY RESPONSE AND LUNG INJURY INDUCED BY Crotalus durissus VENOM" has a descriptive approach about a systemic inflammatory response induced by Crotalus durissus venom associated with aggressive and acute pulmonary injury.

Some minor corrections can be done.

On pdf page 11, line 137: use " with a coupled digital camera " instead of "with an coupled digital camera".

On page 13, line 204: revise Figure 09 citation, this is out of the sequence.

Figure 5 legend need to be corrected: There is text written in Portuguese “Coloração TM, objetiva”

Some Considerations:

1) It is critical to defend systemic inflammatory response without investigate blood sample. Why the authors choose peritoneal lavage instead of serum?
2) The authors consider that the dose administered was lethal (see page 10, lines 113 and 114). Data shown in figure 2 and 3 did not demonstrate a severe and lasting inflammation in both sample used, especially in the lungs, once 3 hours after inoculation was the last time-point with increased pro-inflammatory markers. To consider a lethal dose, authors should provide survival curve.
3) An important deposit of connective tissue is observed around airways. I recommend some morphological quantification to validate that.
